# 

**Published:** 1921-07-23

**Authors:** 


### The Labour Party and Mass Contributions.

The report of Lord Cave's Committee on the voluntary hospitals has been considered by the Labour Party, which suggests that the Government grant should impose the condition that adequate public representation be made on all bodies, including hospital boards, through whose hands the grant passes. So far as hospital boards are concerned the Labour Party's wishes are already secure. Hospitals which have already received regular contributions from workshops have naturally granted representation to the workers, and those now inviting them will equally expect to provide it. The Labour members also seem to fear that the system of patients' payments may secure priority of admission to those able to pay. The fear does not seem well founded, and we believe that there is no occasion for it. The Party's general endorsement of the report of Lord Cave's Committee shows that Labour men also agree that the hospitals' difficulties can best be met by mass contributions.

### Investiture by the King.

At the Investiture held by H.M. the King at Buckingham Palace on July 19, members of the Military and Civil Nursing Services were personally invested by H.M. as follows:—

*Royal Red Cross* (*Bar*).—Matrons Alice Bond, Emily Cox, Lilian Mackay, Gertrude Smith, and Adelaide Walker, all of the Q.A.I.M.N.S.; Matron Henrietta Burton, Q.A.I.M.N.S.R.; Matron Ida Turner, T.F.N.S. *First Class*: Matrons Elizabeth Cooke and Mary Grierson, and Sister Susanna Daly, all of Q.A.I.M.N.S.; Nursing Sister Dorothea West, Q.A.M.N.S., India; Matron Mary Duff, Assistant Matron Mary Sketchley, Sisters Dorothy Black, Marion Leppard, Kathleen O'Reilly, all of Q.A.I.M.N.S.R.; Matron Mabel Allibone, Assistant Matrons Alice Allan and Mary Dando, Sister Mary Mee, Staff Nurse Minnie Byrne, all of the T.F.N.S.; Matron Theodora Bickerton, Civil Nursing Service; Matrons E. Margaret Fox and Ellenor Stevenson, British Red Cross Society; Nursing Sister Kate Sunderland, Civil and War Hospitals.

After the Investiture the above were received at Marlborough House by Queen Alexandra. Miss A. Beadsmore Smith (Matron-in-Chief, Q.A.I.M.N.S.) was also received by Her Majesty.

Miss Agnes Waterhouse, Q.A.M.N.S. (India), was also invested with the insignia of the Order of the British Empire (Commander).

### Presentation to Miss Hoadley.

On the occasion of Miss Hoadley's relinquishment of the office of Lady Superintendent of the Nurses' Co-operation to join her sister in the conduct of a nursing home at Bexhill, an "At Home" was held at 22 Langham Street on July 15, when the nursing staff, through Miss M. E. Murray, presented her with a travelling clock and a cheque. A large number of nurses were present, as well as Mr. Worthington, who attended by his special wish in order to express his appreciation of the services he has received from Co-operation nurses whom he has employed from time to time, and of the help and sympathy rendered at the office by Miss Hoadley. Miss Hoadley, in thanking the staff for their presentation, declared the clock would be a constant reminder of their kindness, while the cheque made her feel quite rich. She was very sad at the thought of parting from the work which had brought her much happiness and pleasure, and she earnestly wished the Co-operation even more success in the future than in the past. Success, she reminded them, depends greatly on carrying out the true spirit of co-operation.

### The Latest Hospital Magazine.

King's College Hospital has now joined the number of institutions with a magazine of more or less general interest. *The King's College Hospital Gazette* is to be a quarterly, of which the first number, dated June, lies before us. It is much to be hoped that old King's men will give the *Gazette* a good start, and spread its reputation in the scattered places where they now live. Its issue is a sign of a post-war revival, which even the recent closing of 160 beds cannot permanently check. The object of re-starting a hospital magazine is not only "to keep the various branches and departments informed of each other's activities," but "to open channels of communication between all who are interested in the work" of the institution. The first number contains a clinical article; a study of Ambroise Paré, the sixteenth-century French surgeon; some humour in prose and in verse; accounts of the clubs and societies, and so forth. We note particularly that the journal is to be "a hospital magazine, and not merely the organ of the medical school." This being so, we hope that the editorial notes will be extended, that the Secretary's department will contribute to them, and that any schemes for raising weekly contributions, a matter in which Mr. R. J. Coles is known to be interested, will be chronicled as they grow. How to interest a wider circle and a local public can be gathered from a study of such London hospital magazines as the Saturday Fund's *Journal* and *Progress*. In regard to general contributions from the hospital's various staffs, if they will only write about what interests themselves they will be interesting to others. We welcome the new paper, and hope that it will flourish in the general concern now being shown, because of their difficulties, in hospital affairs.

### The July "Progress."

The current number of *Progress*, the monthly journal of the Great Northern Central Hospital, Holloway, contains interesting accounts of the hospital's doings and organisations, besides many illustrations. There is a description of Grovelands, Southgate, the home of recovery attached to the hospital, which the Princess Royal opened on July 9; a visit to the children's ward is described by Miss Joan Kennedy, and a full statistical statement is published of the second house-to-house collection held in the spring. It appears that the districts in which the collection is held are organised on the basis of the parishes in the area. During the past year these collections have raised some £4,300. They are held twice a year. Cursory mention is also made of the hospital's efforts to form a working agreement with the four cottage hospitals of North London. The matter is important because of the Hornsey Cottage Hospital's appeal for £20,000 for war-memorial extensions. What working arrangement is proposed in regard to this? As a test case this will serve as well as another.

### The International Conference on Tuberculosis.

At this Conference, which is to be held at the Institute of Civil Engineers, Westminster, from July 26 to 28, over 100 delegates from other countries have announced their intention of being present. M. Léon Bourgeois, President of the French Senate, will be in the chair, and among those who have accepted invitations are Sir George Newman, Sir Robert Philip (President-Elect), Sir Humphrey Rolleston, Hon. Sir Arthur Stanley, Sir St. Clair Thomson, Professor Calmette, Assistant Director of the Pasteur Institute, Colonel Bushnell, head of Medical Department of the U.S.A. Army. Delegates are also coming from Italy, Norway, Sweden, Holland, Belgium, Australia, Canada, South Africa, India, New Zealand, Denmark, Czecho-Slovakia, Greece, Argentina, Brazil, Spain, Roumania, the Republic of Colombia, Guatemala, Luxemburg, Haiti, Monaco, and Panama. Lord Curzon and Sir Alfred Mond, Minister of Health, will welcome the Conference on behalf of the Government.

### The College of Nursing.

**(Limited by Guarantee.)**

### LONDON CENTRE.

(Secretary, Miss Bompas, 7 Henrietta Street, W. 1.)

Tuesday, July 26, 7 to 9 p.m.—The Jumble Sale Committee of the London Centre will be At Home at the Club Rooms, 7 Henrietta Street, W. 1. Music, novel and exciting entertainment, refreshments. Non-members and their friends are invited.

### BIRMINGHAM THREE COUNTIES CENTRE.

(Hon. Secretary, Mrs. Glegg, 84 Hagley Road.)

Tuesday, July 26, 5.30 p.m.—In the Lecture Theatre of the General Hospital, Birmingham (by kind permission of the Governors), Dr. Harries, medical superintendent of the City Hospital, Little Bromwich, will lecture on "Barrier Nursing in Fever Cases." Admission, members free; non-members, 1s.

### YORKSHIRE CENTRE, LEEDS.

(Hon. Secretary, M. V. Lindall, Hospital for Women and Children, Leeds.)

Saturday, July 30, 3.30 to 6 p.m.—Garden-party at Seacroft Hospital, Leeds (by kind invitation of Miss Tomlin, R.R.C., Matron). Killingbeck cars leave City Square or the Barriers, outside the entrance to the Market, every ten minutes. Members wishing to attend the Garden-party are asked to notify Miss Tomlin by Wednesday, July 27, at the latest. Membership cards should be presented on admission.

### Personalia.

Miss Ratcliff, Matron of the Brighton Sanatorium since 1890, has resigned "owing to increasing years." She is now seventy-nine years of age, but, judging from the efforts which the medical officer of health, Dr. Duncan Forbes, has made to retain her services "as an experienced and still active member of the staff," it is evident that her powers are still considerable. She has earned the esteem and confidence of a long series of Health Committees, and has practically devoted her life for over thirty years to her institution. She retires on a pension of £75, "subject to her rendering such assistance at the Sanatorium as she may be called on to perform."

### The New Massage Building at Guy's.

Mr. Wm. J. Walford, F.R.I.B.A., Leadenhall Buildings, E.C., calls our attention to the fact that on the plans of the new massage building at Guy's Hospital, which were reproduced in our issue of July 16, page 262, the scale is stated to be 16 feet to 1 inch. This was the scale to which the original small plans were drawn, and our illustration is a reduction to half the size. We regret that this fact was not mentioned.

### The Bucknall Hospital Litigation.

The Stoke-on-Trent and Stoke Rural Joint Hospital Board have prosecuted the managing director of Hanley Garage, Ltd., and G. J. Stephenson, an engineer, formerly in the Board's employment, on a charge of conspiring, between March 1915 and August 1919, to obtain various sums, amounting to £135, from the Board by falsely pretending that the garage had supplied certain goods to the Hospital Board. As we write the case is being heard at the Staffordshire Assizes.

### Forthcoming Events.

Tuesday, July 26, 4.30 p.m.—Meeting of the London Regional Committee of the British Hospitals Association at Lord Hambleden's house, 3 Grosvenor Place, S.W., to consider (*a*) the question of mass contributions and the conferences that have been called by the King's Fund in consequence of the recommendations in Lord Cave's Report; (*b*) a report on negotiations with the Approved Societies; (*c*) report of a conference with the College of Nursing Salaries Committee on the scale of salaries.

Wednesday, July 27, 3 p.m.—View Day at the Great Northern Central Hospital, Holloway Road, N. The Chairman (the Marquess of Northampton) will receive visitors in the Board Room. The wards can be visited from 3 to 4.30 Music Tea in the Nurses' Garden (weather permitting).

### The Hospital

**RATES OF SUBSCRIPTION** (post free, payable in advance).
Three months, 3/3; six months, 6/6; twelve months, 13/-. (Should the cost of printing and paper as well as increased postage, necessitate alteration of these rates, the period paid for will be reduced proportionately on unexpired subscriptions.) THE HOSPITAL "Index" to Volume LXIX., October 1920 to March 1921, is now ready, price 1/1 per copy, post free. Address The Manager, THE HOSPITAL, "The Hospital" Building, 28/29 Southampton Street, Strand, London, W.C. 2.

### UNIFORM SYSTEM ACCOUNT BOOKS.

A Complete Set of Account Books, ruled in accordance with the Uniform System adopted by the King Edward VII. Hospital Fund for London and the Metropolitan Hospital Sunday Fund. Especially designed and constructed for the convenience and assistance of Secretaries of Hospitals and all classes of Institutions.

### SERIES No. 1.—FOR HOSPITALS AND INSTITUTIONS.

1. ANALYSIS JOURNAL. Royal, 350 pp. Divided into seven sections, with tags. Headings as follows:—
   A. Maintenance.
   1. Provisions.
   2. Surgery and Dispensary.
   3. Domestic.
   4. Establishment and Miscellaneous.
   5. Salaries, Wages, etc.
   B. Administration.
   1. Management, Rent, Rates, and Taxes.
   2. Finance.
   C. Extraordinary Expenditure.
   (Uniform with the New Index of Classification adopted by King Edward VII. Hospital Fund for London.)
   Each Heading is ruled with pages containing 15 columns. Price £3 10s.
2. CASH BOOK. Foolscap, 270 pp., printed Headings, specially ruled. Price £1.
3. CASH ANALYSIS AND RECEIPT BOOK. Foolscap, extra width, 300 pp., ruled with 15 columns, printed Headings identical with Income and Expenditure Table. Price £2 15s.
4. SECRETARY'S PETTY CASH BOOK. 300 pp., foolscap, ruled with 13 columns, printed Headings identical with Income and Expenditure Table. Price £1 7s. 6d.
5. LIST OR REGISTER OF ANNUAL SUBSCRIBERS. Double foolscap, 300 pp., ruled with 10 columns, printed Headings, etc. Price £2 5s.
6. ALPHABETICAL REGISTER BOOK. Foolscap, 300 pp., ruled, with date, name and address, and cash columns, cut into alphabetical sections, with totals at end for annual balance. Price £1 10s.
7. SUBSCRIPTION REGISTER. Foolscap, ruled with 8 columns, printed Headings for date when subscriptions become due, name and address, and cash columns. Price £1 7s. 6d.
8. LINEN REGISTER. Foolscap, long 4to, 300 pp., ruled with columns for Stock, Condemned, Remaining and Issued Linen, also total in Ward and Remarks, also printed Lists of Hospital Linen. Price 12s. 6d.
9. INCOME AND EXPENDITURE TABLE. For yearly balances and statements. Royal. Price 8d. per sheet, or 7s. 6d. per dozen

### SERIES No. 2.—FOR COTTAGE HOSPITALS AND SMALLER INSTITUTIONS.

1. CASH ANALYSIS, RECEIPT AND EXPENDITURE BOOK. Double foolscap, 300 pp., ruled with 18 columns, printed Headings identical with Income and Expenditure Table, specially prepared for Cottage Hospitals and Small Institutions. Price £3.
2. SECRETARY'S PETTY CASH BOOK. 300 pp., foolscap, ruled with 13 columns, printed Headings identical with Income and Expenditure Table. Price £1 7s. 6d.
3. LIST OR REGISTER OF ANNUAL SUBSCRIBERS. Double foolscap, 300 pp., ruled with 10 columns, printed Heading, etc.—Price £2 5s.
4. LINEN REGISTER. Foolscap, 300 pp., ruled with columns for Stock, Condemned, Remaining and Issued Linen, also total in Ward and Remarks, also printed Lists of Hospital Linen. Price 12s. 6d.
5. INCOME AND EXPENDITURE TABLE. For yearly balances and statements. Royal. Price 8d. per sheet, or 7s. 6d. per dozen.

Carriage extra in all cases.

**The Books are all uniformly bound in half basil, with gilt letterings, and are ruled on best paper and in every way prepared for practical use in the offices of Institutions.**

**THE SCIENTIFIC PRESS, LTD.,** 28/29 SOUTHAMPTON STREET, STRAND, LONDON, W.C. 2.

